# Microbicidal Phagocytosis of Nucleus Pulposus Cells Against *Staphylococcus aureus* via the TLR2/MAPKs Signaling Pathway

**DOI:** 10.3389/fimmu.2019.01132

**Published:** 2019-05-22

**Authors:** Yazhou Lin, Hui Cong, Kewei Liu, Yucheng Jiao, Ye Yuan, Guoqing Tang, Yong Chen, Yuehuan Zheng, Jiaqi Xiao, Changwei Li, Zhe Chen, Peng Cao

**Affiliations:** ^1^Department of Orthopedics, Kunshan Hospital of Traditional Chinese Medicine, Kunshan, China; ^2^Shanghai Key Laboratory for Prevention and Treatment of Bone and Joint Diseases With Integrated Chinese-Western Medicine, Ruijin Hospital, Shanghai Institute of Traumatology and Orthopedics, Shanghai Jiaotong University School of Medicine, Shanghai, China; ^3^Department of Oncology, The Affiliated Hospital of Qingdao University, Qingdao, China; ^4^Kunshan Hospital of Traditional Chinese Medicine, Kunshan, China; ^5^Department of Orthopedics, Ruijin Hospital North, Shanghai Jiaotong University School of Medicine, Shanghai, China; ^6^Department of Medical Microbiology and Parasitology, Shanghai Jiaotong University School of Medicine, Shanghai, China

**Keywords:** nucleus pulposus cells, phagocytosis, phagolysosomes, *Staphylococcus aureus*, TLR2, MAPKs

## Abstract

Intervertebral disc (IVD) is an immune-privileged organ that lacks immunocytes, such as macrophages or neutrophils; therefore, it is unclear how IVD immunological defense against bacterial infection occurs. Here, we demonstrated that nucleus pulposus cells (NPCs), the vital machinery for maintaining the homeostasis of IVD, exerted microbicidal activity against *Staphylococcus aureus* via induction of phagolysosome formation. Moreover, we found that the Toll-like receptor 2 (TLR2)/mitogen-activated protein kinases (MAPKs) signaling pathway is critical for bacterial phagocytosis and phagolysosome formation of NPCs. These findings demonstrated for the first time that NPCs could function as non-professional phagocytes against *S. aureus* infection, thereby enhancing antimicrobial defense against bacterial infections in IVDs.

## Introduction

Intervertebral discs (IVDs), which are partially movable joints that connect each of the vertebral bodies in the spine, play a key role in maintenance of the physiological construction of the spine, functioning both to transfer loads and impart mobility (1). However, spondylodiscitis (SD), which is the result of an infection that reaches IVD and/or adjacent vertebral body, epidural space and paraspinal tissue by hematogenous route, direct inoculation or from a contiguous focus (2), is one of the main etiologies resulting in dysfunction and destruction of IVDs (3).

The most frequent bacterium to infect IVDs is *Staphylococcus aureus*, and its prevalence in infection of IVD has been shown to be as high as 80% (4). However, treatment of *S. aureus*-induced SD is difficult because there are so many antibiotic-resistant strains of *S. aureus*, and the IVDs are not easily penetrated by antibiotics (5, 6). Thus, it is necessary to understand the innate protective immune responses against *S. aureus* in IVDs, thereby developing immune-based antibacterial therapies to combat *S. aureus* infection.

The response to bacterial infection includes professional phagocytes, such as macrophages, which defend against invading bacteria not only via the formation of phagosomes (7) but also by presenting antigens to the adaptive immune system (8, 9). However, IVDs, particularly the central nucleus pulposus (NP), are immune-privileged organs with little or no direct vasculature supply; therefore, the accessibility of immunocytes, such as macrophages and neutrophils, is limited to the outer layer of the IVDs (5, 10). As a result, it is not clear how IVDs defend against bacterial infection without the assistance of professional phagocytes.

Previous studies have suggested that NPCs may serve as macrophages to play an immune defensive role (11). In addition, bovine NP cells have been found to phagocytose latex beads and apoptotic bodies, and the pattern of phagocytosis is similar to the committed phagocytes of THP1 and J774 cell lines (10). Unfortunately, there is still no evidence confirming the phagocytic ability of NPCs or whether NPCs have the ability to defend against *S. aureus* in IVDs.

In the present study, we demonstrated that NPCs exerted microbicidal activity against *S. aureus* via induction of phagolysosome formation. Moreover, we found that Toll-like receptor 2 (TLR2)/mitogen-activated protein kinases (MAPKs) signaling pathway activation is critical to bacterial phagocytosis and the phagolysosome formation of NPCs. To our knowledge, this is the first study to investigate the phagocytic and microbicidal function of NPCs, and our findings provide new insight into the innate immunological ability of IVDs against bacterial infection.

## Materials and Methods

### Ethics Statement

All human sample acquisitions were approved by the ethical committee of Ruijin Hospital, SJTU School of Medicine, China, and performed in accordance with the Declaration of Helsinki Principles. All participants provided written informed consent, which was obtained before enrollment in the study. All animal experiments were performed according to the protocol approved by the SJTU Animal Care and Use Committee and in direct accordance with the Ministry of Science and Technology of the People's Republic of China on Animal Care guidelines. All surgeries were performed under anesthesia and all efforts were made to minimize suffering.

### Patients and Tissue Harvesting

A total of 6 patients were included in this study. The patients underwent discectomy at the single-level lumbar spine due to disc degeneration associated with sciatica and/or low back pain, and these patients presented without the symptom of lumbar segmental instability, such as spondylolisthesis. All patients had decided on surgery after failed attempts to improve their condition using conservative treatment for several months. The average age of patients included in the study was 56.78 ± 14.59 years, and 4 patients were male and 2 patients were female. The levels of surgery were as follows: 3 at L4~5, and 3 at L5~S1.

Based on a stringent antiseptic sterile protocol described in our previous study, a posterior discectomy was performed to harvest IVDs (12, 13). Briefly, the skin of the operation field was sterilized 3 times with povidone iodine, and a 3M Ioban 2 Antimicrobial Incise Drape (3M Health Care, St. Paul, MN, USA) was used to cover the surgical field. The wound was then irrigated twice using sterile water before discectomy of the IVDs. The harvested specimen was handled exclusively with sterilized instruments to avoid contamination.

### Bacterial Strains and Growth Conditions

A standard strain of *S. aureus* (ATCC: 25923) was kindly provided by Dr. Xu Chen (Shanghai Ninth People's Hospital, Shanghai Jiaotong University School of Medicine, Shanghai, China). A *S. aureus* strain (NCTC8325) carrying the *gfp* gene was kindly provided by Pro. Bing Hu (University of Science and Technology of China, Hefei, China). The bioluminescent *S. aureus* Xen36 (PerkinElmer, Inc., Waltham, MA) was provided by Dr. Deming Jiang from East China Normal University.

*S. aureus* were cultured overnight until the static phase at 37°C in tryptic soy broth (TSB) medium (Difco, Detroit, Mich., USA). Then, the bacteria in the static phase were pelleted via centrifugation at 10,000 g for 5 min. The supernatant was discarded, and the pellet of cells were resuspended in sterile phosphate buffered saline (PBS), washed twice with a PBS, and suspended in PBS supplemented for late-stage experiments.

### Co-cultures of Nucleus Pulposus Cells (NPCs) and *S. aureus*

Human NP tissues were harvested following the above protocol and the *Tlr*2^−/−^ NP tissues were obtained from *Tlr*2^−/−^ mice IVDs. Cell samples from different patients and mice were kept separate. All experiments were carried out three independent experiments and were conducted with NPCs from passages 2 to 3.

For co-culture, the bacteria were harvested from overnight cultures in stationary phase and washed twice with phosphate-buffered saline (PBS). The bacterial density was adjusted to optical density (OD_600_ = 2). Then, *S. aureus* were added to the cell culture (5 × 10^5^ cells/well) in a 6-well culture plate at a 1:10 multiplicity of infection (MOI) without antibiotics and fetal bovine serum (FBS). After 0, 0.5, 1, 2, and 4 h, co-cultured cells were washed vigorously five times with PBS and prepared for late-stage experiments (including transmission electron microscopy (TEM), bacterial killing assay, Western blot analysis, and confocal laser scanning microscope). In addition to vigorous washing, we have also used lysostaphin to cleave extracellular *S. aureus* (14), as well as quenching the extracellular fluorescence with trypan blue (15).

### Inoculation of *S. aureus* Into Caudal Rat Intervertebral Discs

Eight-week-old male Sprague-Dawley rats were purchased from the Shanghai Laboratorial Animal Center at the Chinese Academy of Sciences. The animals were housed with *ad libitum* access to water and food in an air-conditioned room with a 12-h light-dark cycle, at 21–23°C and 60% relative humidity, in the animal facility at Ruijin Hospital, Shanghai Jiao Tong University School of Medicine, China. Rats were anesthetized intraperitoneally with 2.5% sodium pentobarbital (1.3 mL/kg) and placed in a prone position, with 4 rats per group. Then, the tail skin was sterilized with 75% alcohol three times. Before surgery, the target vertebrae (Co)6/7–(Co)8/9 (*n* = 3 per animal) were identified and marked by palpation and X-ray. The diameters of the target IVDs were measured using X-ray before surgery to determine the depth of puncture. A volume of 2.5 μl *S. aureus* (OD_600_ = 2.0) was inoculated vertically into the nucleus pulposus using a microsyringe with a 28-gauge needle (Hamilton, Nevada, USA). The penetration depth was fixed at 2.0–2.5 mm using a stopper. All surgeries were performed under anesthesia, and all efforts were made to minimize suffering. After inoculation 4 h, the caudal IVDs tissues inoculated with or without *S. aureus* were collected for electron microscopy test.

### Electron Microscopy

For TEM, NPCs infected with *S. aureus* on chamber slides and the caudal rat IVDs infected with *S. aureus* were fixed with 2.5% glutaraldehyde in 0.1 M phosphate buffer (pH 7.4) for 2 h, respectively. Conventional electron microscopy was performed as follows. After five washes with 0.1 M phosphate buffer, the cells were post-fixed with 2% osmium tetroxide and 0.5% potassium ferrocyanide in the same buffer for 1 h and then washed again with 0.1M phosphate buffer. After dehydration, the cells and tissues were embedded in Epon 812 (TAAB Laboratories Equipment Ltd.). Ultrathin sections were stained with uranyl acetate plus lead citrate and observed using an H7700 electron microscope (Hitachi, Tokyo, Japan).

### The Imaging Analysis of Nucleus Pulposus Infected by *S. aureus*

An IVIS 100 instrument (Caliper Life Sciences, Hopkinton, MA) was used to acquire the bioluminescence images. NPCs were infected with bioluminescent *S. aureus* strains (MOI = 1:10) in 6-wells plates at different time (0, 0.5, 1, 2, 4 h) and were then washed five times with PBS for eliminating extracellular *S. aureus*. The 6-wells plates were transferred to the imaging chamber. Bioluminescent images are represented using a pseudocolor scale (with blue representing the least-intense and red representing the most-intense light) that was overlaid on a gray-scale image to generate a picture of the distribution of bioluminescent bacteria in the cells.

NPCs were co-cultured with GFP-labeled *S. aureus* (MOI = 1:10) on glass slides at different time points (0.5, 1, 2, 4 h) and were then washed five times with PBS. *S. aureus*-infected cells were stained for 30 min with phalloidin and 5 min with DAPI at room temperature, respectively.

NP cells were plated on round 12-mm glass coverslips in 24-well tissue culture plates at a density of 1 × 10^5^ NP cells/coverslip and then co-cultured with *S. aureus* (MOI = 1:10) at different time points (0, 0.5, 1, 2, 4 h). After cells were washed five times with PBS, NP cells were treated by lysosomes labeled with 50 nM LysoTracker Red for 60 min then DAPI labeled for 5 min. All images were observed using a fluorescence microscope (Axio, Carl Zeiss, Oberkochen, Germany).

### Bacterial Killing Assay

NP cells were plated at 5 × 10^5^ cells/well in 6-wells plates. *S. aureus* were added at a multiplicity of infection (MOI) of 10 and co-cultured 1 h. Each well was washed 5x with ice-cold PBS to remove extracellular bacteria, as well as cleaving extracellular *S. aureus* with lysostaphin and quenching the extracellular fluorescence with trypan blue. To measure bacterial uptake at the end of 1 h, triplicate wells were lysed after washing in 1 ml sterile water. This was designated as time 0. Warm medium was added to the remaining wells, and the cells were placed in cell incubator for additional times of 0.5, 1, 2, and 4 h. At each time point, triplicate wells were washed 5x with ice-cold PBS before lysing the cells. Viable counts were determined by gradient dilution method. The different dilution (1:10^2^, 1:10^3^, 1:10^4^, and 1:10^5^) add to agar LB medium. The intracellular *S. aureus* was determined by counting the number of colony forming units (CFU) at each time point and was calculated by Log (#CFU at time X).

### Flow Cytometry Analysis

NP cells (WT or *Tlr2*^−/−^, 5 × 10^5^ cells/well in 6-wells) were incubated in F12-DMEM without FBS containing either the carboxylate-modified latex beads (2%0, No. L3030, Sigma, St. Louis, Missouri, United States) or *S. aureus*-GFP for the different time (0, 0.5, 1, 2, 4 h) in the presence or absence of Cochicin (Phagocytosis inhibitor, 2.5 mM, C3915, Sigma, St. Louis, Missouri, United States), and then each well was washed 5x with ice-cold PBS, lysostaphin, and trypan blue to remove excess beads and *S. aureus*. The internalized fluorescent red latex beads were measured by flow cytometry. The percentage decrease or increase of phagocytosis was calculated.

### Western Blot Analysis

For Western blot analysis, total proteins from the samples were separated by SDS-PAGE, transferred to nylon membranes and incubated separately with the following primary antibodies: MAPKs family kit/P-MAPKs family kit (dilution of 1: 1000; cat. no. 9926T/9910T, CST, Inc., MA, USA). B-actin (dilution of 1: 2000; cat. no. CW0096, CW BIO, Beijing, China) was used as an internal control. Then, the membranes were incubated with horseradish peroxidase-conjugated secondary antibody, goat anti-rabbit IgG (dilution, 1: 2000; cat. no. CW0103s; CW Bio, Beijing, China) or goat anti-mouse IgG (dilution, 1: 2000; cat. no. CW0102s; CW Bio, Beijing, China) at room temperature for 2 h, and the bands were visualized using chemiluminescence (Pierce Biotechnology, Inc., IL, USA). The images were analyzed using Fusion FX7 (Vilber Lourmat, Marne-la-Vallée, France).

### Statistical Analysis

Data were collected from three or more independent experiments and expressed as the mean ± S.D. A Two-sided Student's *t*-test was used to analyze differences between two groups. One-way analysis of variance was performed to show differences among multiple groups. *P* < 0.05 was considered significantly different.

## Results

### Phagocytosis of *S. aureus* by NPCs *in vivo* and *in vitro*

**Figure 1:**
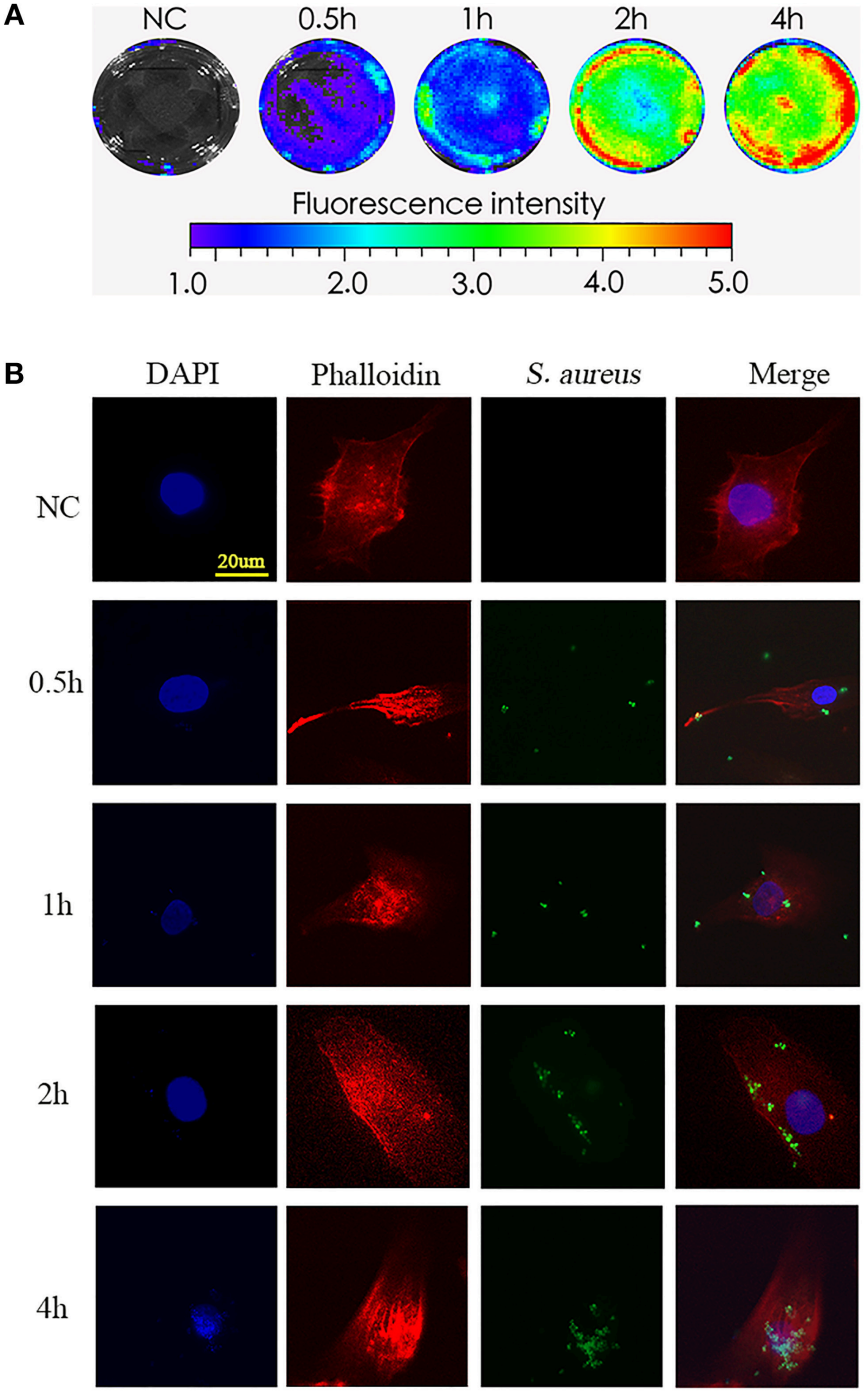
Phagocytosis of *S. aureus* by NPCs *in vitro*. **(A)** NPCs were incubated with bioluminescent *S. aureus* strains (MOI = 1:10) for different time points (0, 0.5, 1, 2, 4 h), and the bioluminescent intensity was detected using a Xenogen-Caliper IVIS-100 instrument. **(B)** NPCs were analyzed by confocal microscopy after incubation with GFP-labeled *S. aureus* for different periods at a MOI = 1:10. The scale bar represents 20 μm. All micrographs are represented at least three experiments.

To test the phagocytosis of NPCs *in vitro*, we incubated NPCs with bioluminescent *S. aureus* strains (MOI = 1:10) for different times (0, 0.5, 1, 2, 4 h), after which the bioluminescent intensity was detected using a Xenogen-Caliper IVIS-100 instrument as previously described (16). As shown in Figure 1A, the bioluminescent intensity increased gradually in a time-dependent manner (Figure 1A), indicating that the interactions between *S. aureus* and NPCs, including the attachment of *S. aureus* to NPCs and internalization, might have occurred during co-culture. To further validate the *S. aureus*-NPCs interaction, NPCs were incubated with the GFP-labeled *S. aureus* and observed by confocal fluorescence microscopy. Bacteria with green fluorescence were found in the cytoplasm area of the NPCs and their numbers increased gradually during incubation, indicating that the bacteria might have been ingested (Figure 1B).

**Figure 2:**
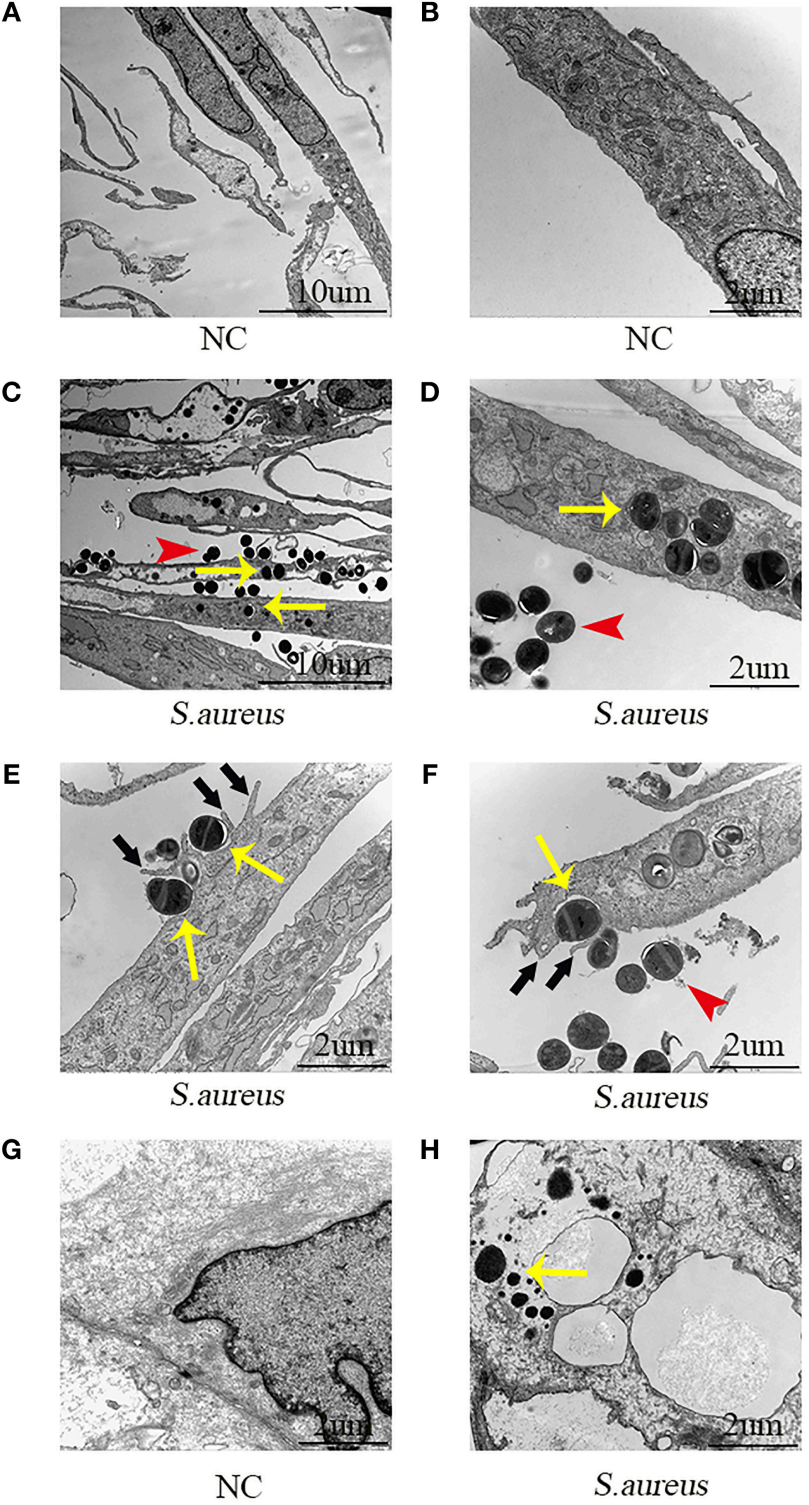
Electron microscopic images of NPCs phagocytizing *S. aureus in vivo* and *in vitro*. **(A,B)** NPCs without incubation with *S. aureus* were characterized by a large rod nucleus and a thin rim of agranular cytoplasm. **(C,D)** After co-culturing the NPCs with *S. aureus* for 4 h, ingested *S. aureus* **(yellow arrow)** was observed in the phagosome within the cytoplasm of NPCs and extracellular *S. aureus* was found **(red arrow**). **(E,F)** There were a varying number of membrane protrusions **(black arrows)** tightly engulfing *S. aureus* **(yellow arrows). (G,H)** Compared with NPCs without incubation with *S. aureus*, ingested *S. aureus* **(yellow arrow)** were observed in the cytoplasm of NPCs after caudal IVD infection with *S. aureus* for 4 h *in vivo*. All micrographs are represented at least three experiments.

Next, the role of NPCs in phagocytosis was evaluated by TEM. The NPCs without *S. aureus* infection were characterized by a large rod nucleus and a thin rim of agranular cytoplasm (Figures 2A,B). After co-culturing the NPCs with *S. aureus* for 4 h, ingested *S. aureus* were observed in the phagosome within the cytoplasm of NPCs (indicated by yellow arrow in Figures 2C–F), and a varying number of membrane protrusions tightly engulfing *S. aureus* (indicated by black arrows in Figures 2E,F), typical of the zipper-like phenomenon of receptor-mediated phagocytosis, were observed. Whereas, extracellular *S. aureus* also were indicated by red arrows in Figures 2C–F. These results demonstrated that NPCs played a role in phagocytosis as indicated by the internalization of *S. aureus in vitro*.

Subsequently, the phagocytosis of NPCs was evaluated *in vivo* using the caudal IVD infection model (17). Compared with the NPCs without incubation with *S. aureus* (Figure 2G) intracellular *S. aureus* were observed in the cytoplasm of the NPCs (Figure 2H) after IVDs were inoculated with *S. aureus* for 4 h. These findings suggested that NPCs had phagocytic ability *in vivo*.

### Microbicidal Activity of NPCs via Phagolysosome Formation

**Figure 3:**
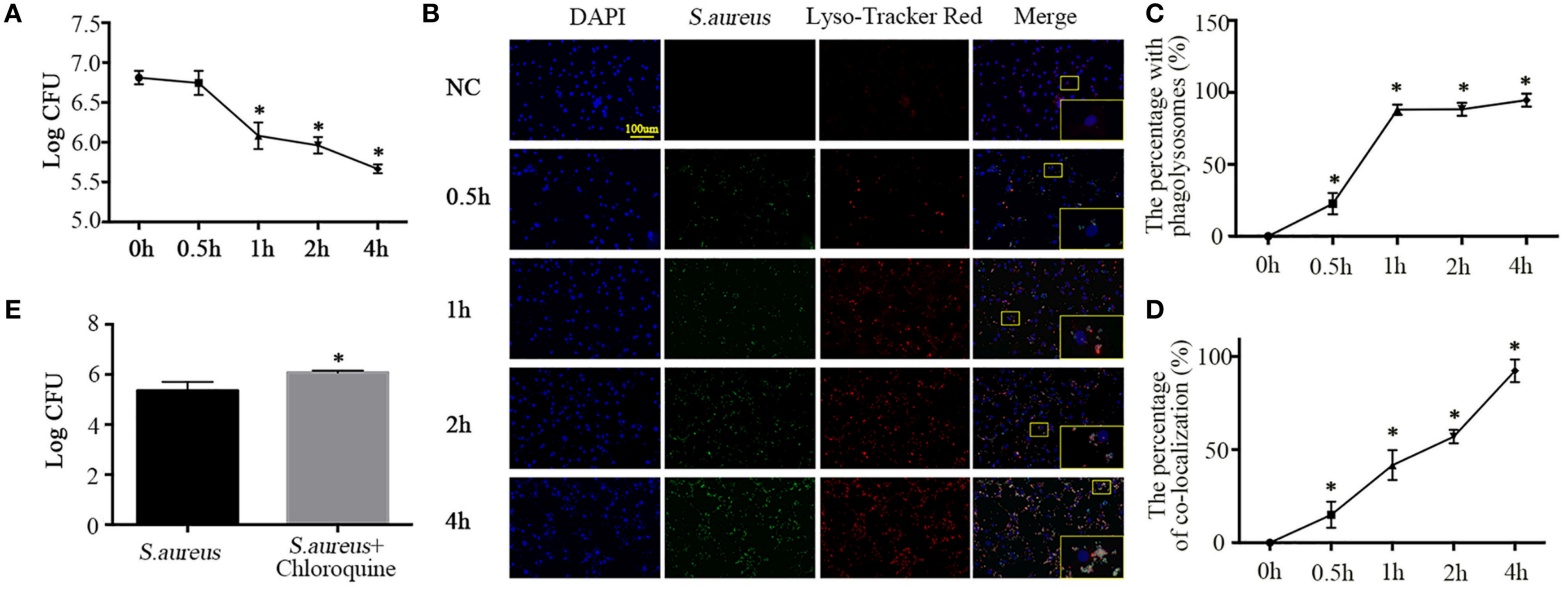
Phagolysosome formation and microbicidal activity of NPCs. **(A)** Intracellular bacterial survival detected by plate counting. The bacterial survival rate is represented as the log CFU/mL. *P < 0.05, group of different time points vs. the group of 0 h. Groups were compared by one-way ANOVA. **(B)** Immunofluorescence staining of Lyso-Tracker Red in NPCs infected with *S. aureus* for different time points (0, 0.5, 1, 2, 4 h). **(C,D)** The percent with phagolysosomes and the percent of co-location increased gradually over time. *p < 0.05, the 0 h group vs. the different groups. Groups were compared by one-way ANOVA. **(E)** Intracellular bacterial survival detected by plate counting. NPCs were induced by *S. aureus* at a MOI = 1:10 for 1 h after pretreatment with chloroquine (25 μM) for 6 h, after which unattached *S. aureus* were removed by washing with ice-cold PBS, lysostaphin, and trypan blue, and then warm F12-DMEM medium was added. The intracellular bacterial survival was determined after 4 h. *P < 0.05, the group of *S. aureus* + chloroquine vs. the group of *S. aureus*. *P* values were determined by *T*-test. All data are presented as the means ± S.D from three independent experiments.

Having established the phagocytotic role of NPCs *in vivo* and *in vitro*, we sought to detect the phagocytic microbicidal activity of NPCs. *S. aureus* were incubated with NPCs at a multiplicity of infection (MOI) of 10, after which internalization was allowed to occur at 37°C in 5% CO_2_ for 1 h and unattached *S. aureus* were removed by washing with ice-cold PBS, lysostaphin (20 ug/ml, L4402, Sigma, St. Louis, Missouri, United States), and trypan blue (1.2 mg/ml, 302643, Sigma, St. Louis, Missouri, United States). Next, warm F12-DMEM medium was added and the cells were placed in cell incubator for additional times of 0.5, 1, 2, and 4 h. After lysing the cells, the intracellular bacterial survival was determined. As shown in Figure 3A, enumeration of bacterial colonies revealed that viable *S. aureus* were gradually killed by NPCs (Figure 3A) in a time-dependent manner.

Ingestion of bacteria by professional phagocytes usually triggers the fusion of phagosomes with primary lysosomes, resulting in the formation of phagolysosomes (18), which contributes to pathogen killing and disruption of microbial components (19). Hence, to further verify whether the NPCs, like professional phagocytes, could activate the fusion of phagosomes and lysosomes to kill the ingested *S. aureus*, we analyzed secondary lysosomes formation using Lyso-Tracker Red (Molecular Probes), which labels late endosomes and lysosomes. We then monitored the maturation of phagosomes containing *S. aureus*–GFP by their ability to co-localize with Lyso-Tracker Red. The results showed that the number of cells positive for Lyso-Tracker Red and ingested *S. aureus* increased gradually with incubation time, as did the percentage of co-localization (Figures 3B–D).

To further investigate the role of lysosomes in NPC killing of intracellular *S. aureus*, NPCs were pretreated with chloroquine (25 μM, No. S6628, Sigma, St. Louis, Missouri, United States), an inhibitor of lysosomes, for 6 h (20). Interestingly, we found that chloroquine pretreatment significantly decreased the microbicidal activity of NPCs (Figure 3E). These findings revealed that ingestion of *S. aureus* by NPCs induced the phagolysosomes formation and suggested that the co-localization of *S. aureus* and secondary lysosomes was responsible for the microbicidal activity of NPCs.

### Regulation of Bacterial Phagocytosis and Phagolysosome Maturation of NPCs via the TLR2/MAPKs Pathway

**Figure 4:**
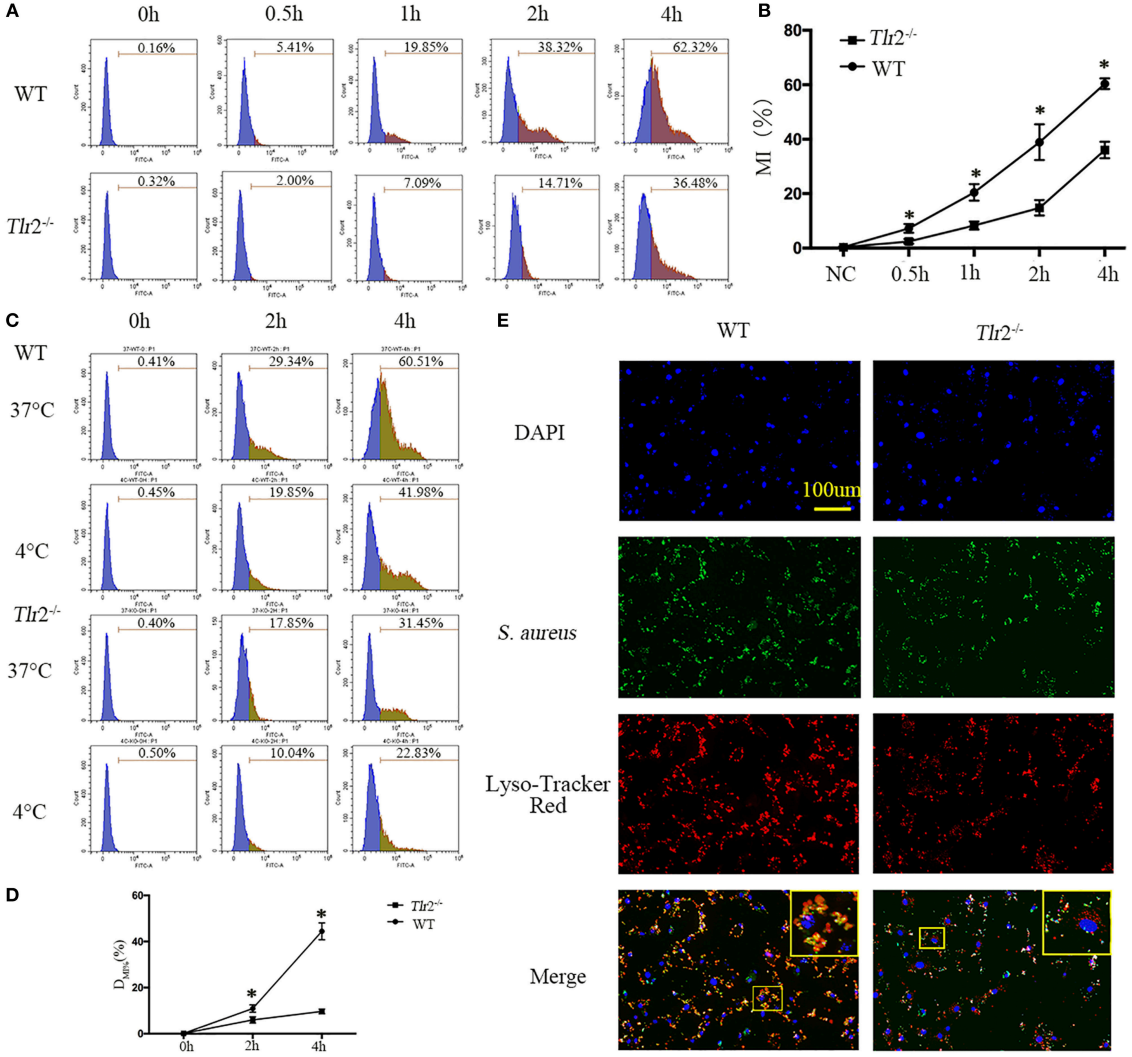
TLR2 regulates the phagocytosis and phagosome maturation of NPCs. **(A,B)** GFP-labeled *S. aureus* were incubated with NPCs from wild-type (WT) and *Tlr2*^−/−^ mice, after which the interaction was compared by flow cytometry analysis. MI% represents mean intensities percentage. *P < 0.05, the group of WT vs. the group of *Tlr2*^−/−^. **(C,D)** The mean intensities percentage (MI%) at 4°C (phagocytosis was inhibited at 4°C) and 37°C were analyzed, respectively. A significant difference in D_MI%_ (MI%_37°C_-MI%_4°C_) was observed in NPCs from WT mice. *P < 0.05, the group of WT vs. the group of *Tlr2*^−/−^. **(E)** Phagolysosomes formation decreased significantly in *Tlr2*^−/−^ NPCs compared with WT NPCs based on Lyso-Tracker Red staining. The scale bar represents 100 μm. *P*-values were determined by *T*-test and one-way ANOVA. All data are presented as the means ± S.D from three independent experiments.

Effective clearance of pathogenic microorganisms by phagocytes involves their initial detection through surface receptors followed by uptake and killing. As widely expressed pattern recognition receptors (PRR) in mammals, TLR2 has been suggested to play a role in modulating the phagocytosis of serials of bacteria, including *S. aureus*, by macrophages and some non-professional phagocytes (21–23). Therefore, to examine whether TLR2 is critical for phagocytosis of *S. aureus* by NPCs, GFP-labeled *S. aureus* were incubated with NPCs from wild-type (WT) mice and *Tlr*2^−/−^ mice, and the uptake of live GFP-*S. aureus* was compared by flow cytometry analysis. The kinetics of interactions between *S. aureus* and NPCs were significantly decreased in the absence of *Tlr*2, as determined by GFP fluorescence intensity (Figures 4A,B). Next, to determine if the decreased interaction was mainly because of decreased intracellular ingestion or surface adherence, fluorescence was analyzed from WT and *Tlr*2^−/−^ NPCs after incubation for 2 and 4 h at 37°C, or at 4°C (because phagocytosis were inhibited at 4°C, MI%_4°C_ represent the surface adherence of *S. aureus*) with GFP-*S. aureus*. As shown in Figures 4C,D, a significant increase in D_MI%_ (MI%_37°C_-MI%_4°C_), which could reflect the phagocytosis of NPCs, was observed in NPCs from WT, rather than in *Tlr*2^−/−^ mice (Figures 4C,D). These findings indicated that internalization, rather than adherence, was responsible for the differences in interactions between WT and *Tlr*2^−/−^ NPCs.

In professional phagocytes, phagocytosed bacteria are initially contained within phagosomes that mature into phagolysosomes (24, 25). Therefore, we investigated whether TLR2 was also involved in phagosome maturation of NPCs. Consistent with the dampened phagocytosis activity, *S. aureus* had already co-localized with Lyso Tracker in the majority of WT NPCs, but only in 40–50% of *Tlr*2^−/−^ NPCs (Figure 4E). Taken together, these data demonstrated that TLR2 is critical for the regulation of bacterial phagocytosis and phagolysosome maturation of NPCs.

**Figure 5:**
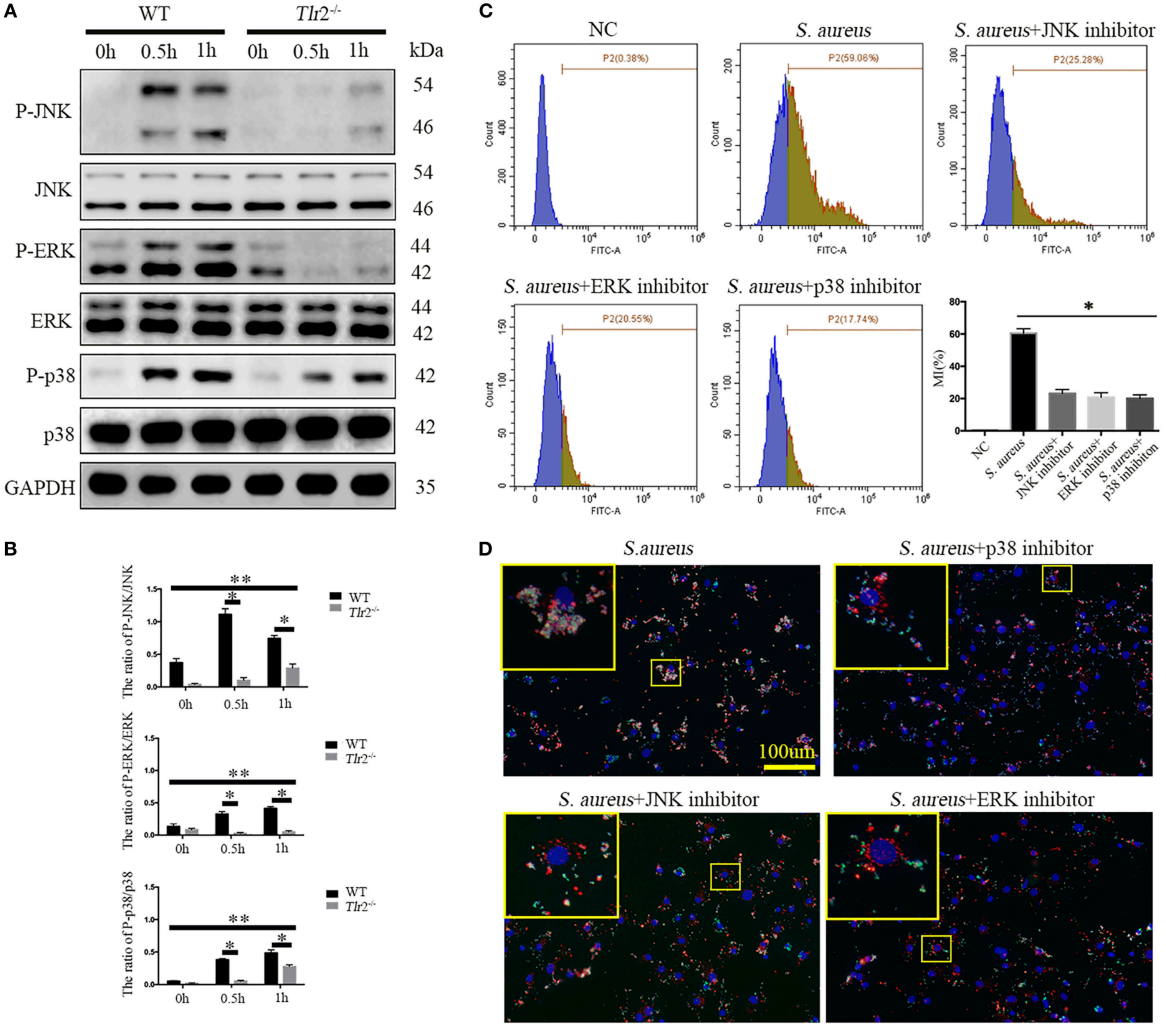
MAPKs are the downstream pathway of TLR2 critical for phagocytosis and phagosome maturation of NPCs. **(A,B)** Western blot analysis of MAPKs (ERK, JNK, p-38) in NPCs from WT and *Tlr2*^−/−^ mice infected with *S. aureus* for different time periods. * *P* < 0.05, the group of WT vs. the group of *Tlr2*^−/−^, **P < 0.05, the different time point in WT group or *Tlr2*^−/−^ group. **(C)** FACS analysis to detect the interaction between NPCs and *S. aureus* with or without MAPKs specific inhibitors pretreatment. *P < 0.05, the *S. aureus* group vs. the different inhibitors group. **(D)** Co-localization of *S. aureus* and Lyso-Tracker Red was detected by fluorescence micrograph. *P* values were determined by *T*-test and one-way ANOVA. All data are presented as the means ± S.D from three independent experiments.

Next, we further explored whether TLR2-mediated downstream MAPKs pathways, including p38MAPK, JNK, and ERK, were involved in bacterial phagocytosis and phagolysosome maturation of NPCs. Phosphorylation of p38 (P-p38), JNK, and ERK in WT NPCs increased dramatically after *S. aureus* infection for 0.5 h, peaking at 1 h (P-JNK peaked at 0.5 h). However, unchanged phosphorylation of ERK and delayed phosphorylation of JNK and p38 MAPK were observed in *Tlr2*^−/−^ NPCs in response to *S. aureus* incubation (Figures 5A,B). The interaction of *S. aureus* and NPCs was significantly impaired following inhibition of p38 MAPK, JNK, and ERK with the corresponding inhibitors, SB203580 (10 μM, no. S1863, Beyotime, Shanghai, China), SP600125 (20 μM, no. S1876, Beyotime, Shanghai, China), and U0126 (20 μM, no. S1901, Beyotime, Shanghai, China), respectively (Figure 5C). Furthermore, the three specific inhibitors markedly impaired the ability of NPCs to efficiently phagocytose *S. aureus*, as no significant co-localization of *S. aureus* and Lyso-Tracker was detected in the presence of inhibitors treatment. These findings indicated a block in phagosome maturation (Figure 5D). Taken together, these results indicate that the TLR2-MAPKs pathway is essential for bacterial phagocytosis and phagolysosome maturation of NPCs.

## Discussion

In the immune system, phagocytosis is the first line response of immunocytes to invading bacteria. Professional or dedicated phagocytes consist primarily of polymorphonuclear neutrophils, monocytes, monocyte-derived macrophages, and tissue-resident macrophages (26). However, in many tissues, there are various types of non-professional phagocytes, such as fibroblasts and glomerular mesangial and endothelial cells (27, 28). Here, it is confirmed for the first time that NPCs, the core cell populations regulating and maintaining the function and homeostasis of IVDs, also have the ability to phagocytize bacteria. Furthermore, NPCs exerted microbicidal activity against *S. aureus* via induction of phagolysosome formation. Mechanistically, activation of the TLR2/MAPKs signaling pathway is critical to bacterial phagocytosis and the phagolysosome formation of NPCs. Therefore, the identification of NPCs as a phagocyte, and elucidation of the role of the TLR2/MAPKs pathway in phagolysosome formation provides a new mechanism for understanding the function of NPCs in defense against bacterial IVD infections.

The IVD is an immune-privilege organ with little or no direct vasculature, particularly the central nucleus pulposus (NP) (5); therefore, the accessibility of immunocytes is likely to be more limited than in most tissues (10). However, it has been shown that there are cells with a phagocytic phenotype in human non-herniated discs. These cells have a similar appearance to NP cells (11) and are capable of behaving as phagocytes to ingest apoptotic cells and latex beads (10). However, whether NPCs could operate as professional non-phagocytes to phagocytize invading bacteria is less known. Here, the ingested *S. aureus* was observed in the phagosome within the cytoplasm of NPCs, and a varying number of membrane protrusions tightly engulfing *S. aureus* typical of the zipper-like phenomenon of receptor-mediated phagocytosis were observed. Our results also revealed that the phagocytosis percentage of NP cells increased dramatically after incubation with microspheres for 1 h, peaking at 4 h (Supplemental Figure 1A). Furthermore, the phagocytosis inhibitor colchicine significantly reduced the NPC phagocytosis (Supplemental Figure 1B). Thus, these results demonstrated the phagocytosis role of NPCs in the internalization of *S. aureus* and microspheres, and that NPCs could be considered “non-professional phagocytes” in IVDs.

The destiny of most phagocytized bacteria is to be eradicated via phagolysis. Generally, after the formation of phagosomes, the maturing process starts immediately, after which it undergoes sequential fusion with early endosomes, late endosomes and lysosomes (29). The terminal stage of the maturation sequence is the formation of phagolysosomes that are endowed with a complete, sophisticated armamentarium to eliminate and degrade microorganisms (30). In the present study, we detected an effectively phagocytic microbicidal activity against intracellular *S. aureus* of NPCs. In addition, significantly increased mature phagolysosomes formation was found in response to *S. aureus* infection, and the number of formed phagolysosomes was negatively related to the number of surviving intracellular bacteria. However, when phagocytosis was inhibited by chloroquine, an inhibitor of lysosomes, the survival of *S. aureus* was significantly rescued. Therefore, our study demonstrated the phagocytic microbicidal activity of NPCs with phagolysosome formation.

In this study, we found that TLR2 was critical and necessary for the phagocytosis of *S. aureus* by NPCs. As a PRR recognizing the pathogen-associated molecular pattern (PAMP) of Gram-positive bacteria (31), TLR2 was confirmed to participate in various immune activities of NPCs in response to bacterial stimulation, such as regulation of cellular autophagy and apoptosis, secretion of inflammatory factors and mediation of the ROS reaction (17). Moreover, the crucial roles of TLR2 in phagocytosis have been verified (21, 22). Here, the positive percentage of intercellular GFP-labeled *S. aureus* in *Tlr*2^−/−^ NPCs significantly decreased compared with WT NPCs, but the phagocytosis of microspheres in *Tlr*2^−/−^ NPCs was not impaired significantly (Supplemental Figure 1C). Therefore, TLR2 plays a key role in phagocytosis of *S. aureus* in NPCs but is not necessary for the phagocytosis of other materials.

Once TLR2 is engaged, some signaling pathways are triggered, such as MAPK family proteins (p38, ERK, and JNK) (32). MAP kinases transduce signals that are involved in a multitude of cellular pathways and functions, such as autophagy, apoptosis, and cell differentiation (33). Moreover, previous studies verified that TLR2 trigging MAPKs played a key role in phagocytosis of *S. aureus* in different cells. For example, TLR2 mediates phagocytosis through the JNK signaling pathway in *S. aureus*-stimulated RAW264.7 cells (23), while p38 and ERK1/2 are involved in *S. aureus* internalization into bovine mammary epithelial cells (34). In this study, the results of Western blotting and co-localization of *S. aureus* and lysosomes demonstrated that activation of MAPKs is involved in TLR2-induced phagosome maturation in NPCs.

In the present investigation, we only focused on the phagocytosis of NPCs for *S. aureus* because it is the most frequent and harmful bacterium affecting IVDs. However, our previous study reported that there were still low-virulence anaerobic bacteria, especially *P. acnes*, latently residing in IVDs, with the prevalence ranging from 13 to 44% (35–39). We found that NPCs are also capable of phagocytizing *P. acnes* under TEM observation according to our previous data (13). These data suggest that the phagocytic ability of NPCs may be universal and not specific; however, further investigation is needed to confirm this.

It should be noted that this study had several limitations. Although our studies demonstrated TLR2 play a crucial role in the phagocytosis activities of NPCs against *S. aureus*, it is unclear if other classical phagocytic receptors, such as Dectin-1/2, Fc receptors or complement receptors (CRs), are also involved in the phagocytosis of NPCs for bacteria. Second, as a Gram-positive bacteria, *S. aureus* was able to initiate TLR2 pathway-mediated inflammatory responses via its major cell wall constituents, such as peptidoglycan (PGN) and lipoteichoic acid (LTA) (40), therefore, which molecules from *S. aureus* triggered the phagocytosis of NPCs is still needed for further investigation.

In conclusion, this study provides new evidence that NPCs could function as non-professional phagocytes and illustrated that the microbicidal phagocytosis of NPCs against *S. aureus* occurs via the TLR2/MAPKs signaling pathway. These results provide new insights into immune function of IVDs and may ultimately lead to the development of novel treatment regimens for discogenic infection.

## Ethics Statement

All human sample acquisitions was carried out in accordance with the recommendations of the ethical committee of Ruijin Hospital, SJTU School of Medicine, China with written informed consent from all subjects. All subjects gave written informed consent in accordance with the Declaration of Helsinki. The protocol was approved by the ethical committee of Ruijin Hospital, SJTU School of Medicine, China.

All animal experiments were performed according to the protocol approved by the SJTU Animal Care and Use Committee and in direct accordance with the Ministry of Science and Technology of the People's Republic of China on Animal Care guidelines. All surgeries were performed under anesthesia and all efforts were made to minimize suffering.

## Author Contributions

PC, ZC, CL, and YL conceived and designed the experiments. YL, HC, and KL performed immunofluorescence staining and confocal imaging. HC, YJ, and YL performed electron microscope studies and Western blotting. YZ, JX, and YL performed bacterial killing assay and flow cytometry analysis. YY, GT, YC, and YL performed animal studies and provided experimental materials and reagents. YL and HC interpreted the data and wrote the manuscript with input from all authors.

### Conflict of Interest Statement

The authors declare that the research was conducted in the absence of any commercial or financial relationships that could be construed as a potential conflict of interest.

## Supplementary Material

Supplemental Figure 1 NPCs phagocytize microspheres. **(A)** The phagocytosis rate increased gradually in a time-dependent manner. **(B)** In the presence of colchicine, the phagocytic ability of NP was significantly restored. **(C)** The phagocytosis rate at 2 h after phagocytosis of microspheres by WT or *Tlr2*^−/−^ NPCs.
