# Supplementary figures and images for: Microbicidal Phagocytosis of Nucleus Pulposus Cells Against Staphylococcus aureus via the TLR2/MAPKs Signaling Pathway
Source: Front Immunol. 2019 May 22;10:1132. doi: 10.3389/fimmu.2019.01132 (PMC6538773; doi:10.3389/fimmu.2019.01132)

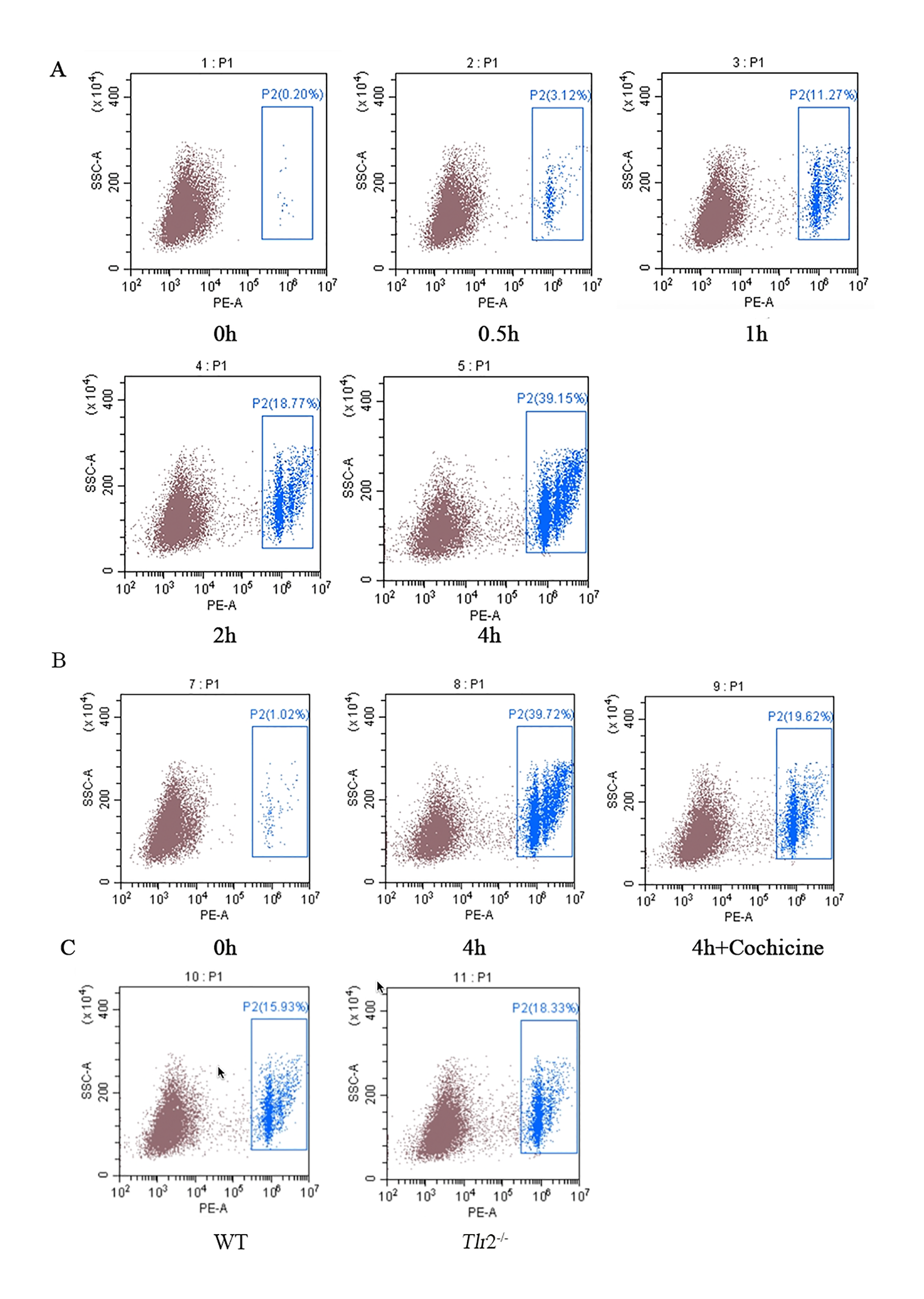

Supplement: Supplemental Figure 1 — NPCs phagocytize microspheres. (A) The phagocytosis rate increased gradually in a time-dependent manner. (B) In the presence of colchicine, the phagocytic ability of NP was significantly restored. (C) The phagocytosis rate at 2 h after phagocytosis of microspheres by WT or Tlr2−/− NPCs. [file Image_1.TIF]
